# Range extension of *Brathinus satoi* in China (Coleoptera, Staphylinidae)

**DOI:** 10.3897/zookeys.344.5740

**Published:** 2013-10-22

**Authors:** Liang Tang, Li-Zhen Li

**Affiliations:** 1 Department of Biology, Shanghai Normal University, 100 Guilin Road, 1st Educational Building 323 Room, Shanghai, 200234 P. R. China

**Keywords:** Coleoptera, Staphylinidae, *Brathinus satoi*, China

## Abstract

*Brathinus satoi* Kishimoto & Shimada, 2003 is recorded from Longwangshan Nature Reserve, Zhejiang, China. Some diagnostic characters of the species are discussed based on more specimens, and some biological notes are made on the species.

## Introduction

*Brathinus* LeConte is a genus belonging to the Omaliinae. Bearing very long elytra covering most abdominal segments, adults of *Brathinus* are pretty bizarre, though their larvae (Thayer 1985) are rather typical Anthophagini. The adults could be misidentified as members of the Anthicidae or Staphylinidae: Scydmaeninae at first glance. In fact, *Brathinus* was originally described as a scydmaenid (LeConte 1852), later placed by LeConte (1861) in its own subfamily of Silphidae (sensu latissimo!), where it was when Lewis (1886) described the first Asian species, and treated as the family Brathinidae by Arnett (1963). Crowson (1955) first placed it in Omaliinae (or at any rate gave no earlier citation) and Hammond (1971) and Thayer (1985) provided more details and justification for that placement.

Presently, six species of the genus have been described: *Brathinus californicus* Hubbard, 1894, *Brathinus nitidus* LeConte, 1852 and *Brathinus varicornis* LeConte, 1852 from North America, *Brathinus oculatus* Lewis, 1886 and *Brathinus shikokuensis* Watanabe & Sato, 1981 from Japan and *Brathinus satoi* Kishimoto & Shimada, 2003 from China. The last species is the only species known from China, and was described from a single male specimen collected from Sichuan. More specimens of *Brathinus satoi* were collected after many years of field work in Longwamgshan Nature Reserve of Zhejiang Province, which is more than 1000 kilometers from its type locality. Thus, some complementary comments on diagnostic characters and biological notes of the species can be provided.

## Material and methods

For examination of the male genitalia, the last three abdominal segments were detached from the body after softening in hot water. The aedeagi, together with other dissected pieces, were mounted in Euparal (Chroma Gesellschaft Schmidt, Koengen, Germany) on plastic slides. Photos of sexual characters were taken with a Canon G7 camera attached to an Olympus SZX 16 stereoscope; habitus photos were taken with a Canon macro photo lens MP-E 65 mm attached to a Canon EOS40D camera. All the specimens treated in the paper were deposited in Department of Biology, Shanghai Normal University, P. R. China.

## Taxonomy

### Brathinus satoi

Kishimoto & Shimada, 2003

http://species-id.net/wiki/Brathinus_satoi

Figs 1 – 9

#### Material examined.

**China: Zhejiang:** 1♀, Longwangshan, 1200m, 25.IV.2004, Jia-Yao Hu leg.; 1♂, Longwangshan, Qianmutian, 1300m, 24.V.2009, Feng & Yin leg.; 11♂♂ 5♀♀, same locality, 1250m–1450m, 30°23'N, 119°26'E, 14.V.2013, Yu, Li, Zheng, Chen, Pan, Hu & Tang leg.; 1♂, same locality, 1050–1200m, near 30°24'28"N, 119°26'25"E, 15.V.2013, Chen & Pan leg.

#### Distribution.

China (Sichuan, Zhejiang).

#### Comments.

**Figures 1–4.**
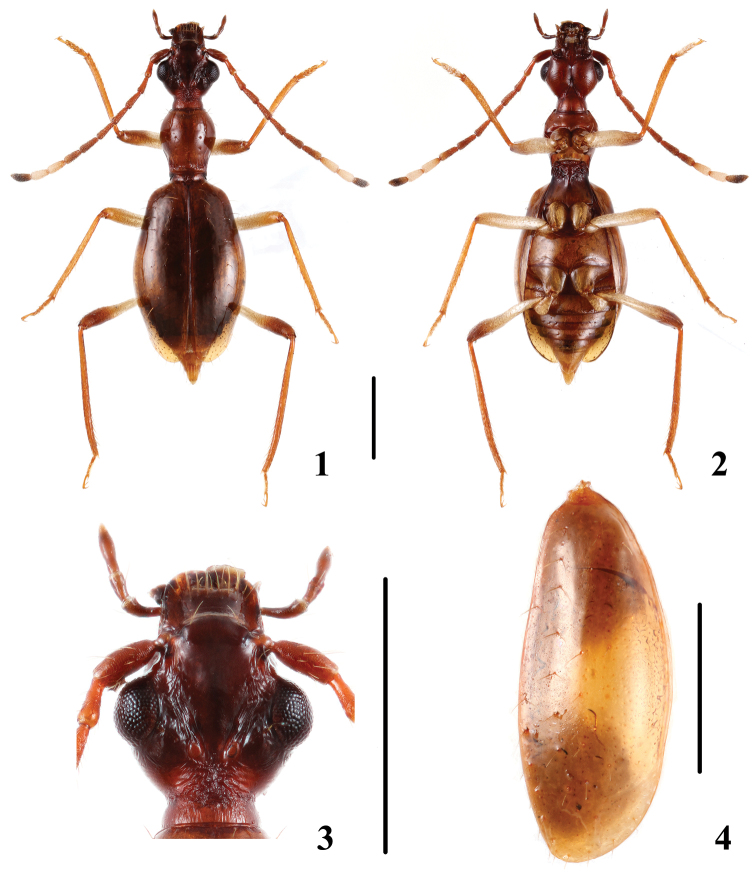
*Brathinus satoi*. **1, 2** adult habitus, (1) dorsal, (2 ventral **3** head, dorsal **4** rightelytron, dorsal. Scale lines = 1 mm.

**Figures 5–7.**
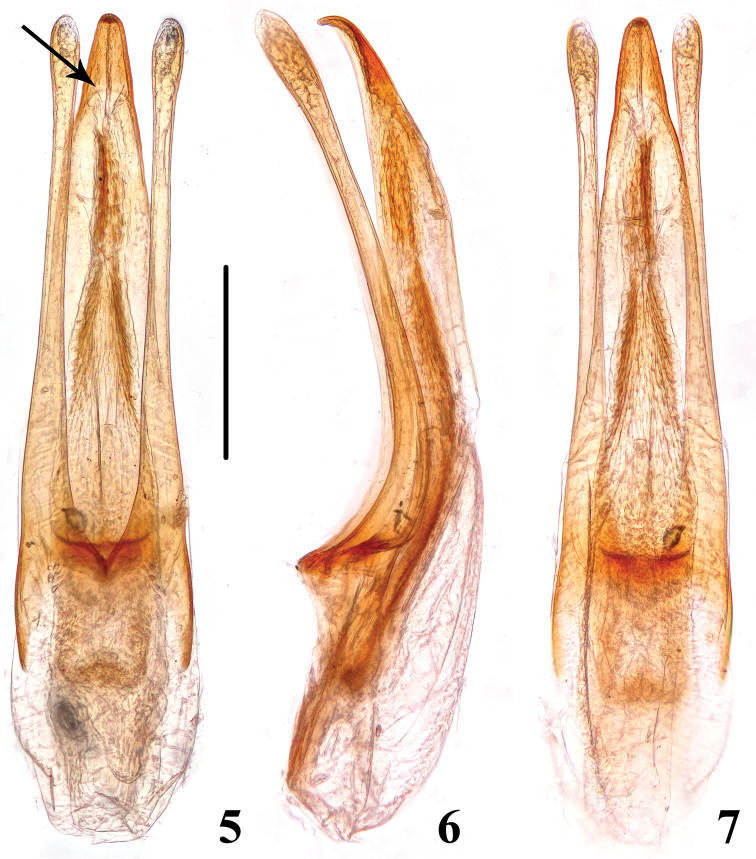
Aedeagus of *Brathinus satoi*. **5** ventral **6** lateral **7** dorsal. Scale line = 0.25 mm.

This species can be easily recognized by rugose punctation along supraorbital furrows (Fig. 3) and several additional characters: antennae reddish brown with antennomeres 9 and 10 pale and antennomere 11 blackish; each elytron (Fig. 4) with a large yellowish mark extending from the elytral center to a broad yellowish band along the lateral margin, anterad and posterad from the midpoint; profemur and metafemur with approximately half of the apical portion darker, mesofemur with apical portion slightly darker (Figs 1, 2); median lobe of aedeagus with a sclerotized apical portion which is delimited basally by a curved margin (Figs 5–7). In immature specimens, however, the elytral coloration is hardly discernible.

#### Biological notes.

**Figures 8, 9.**
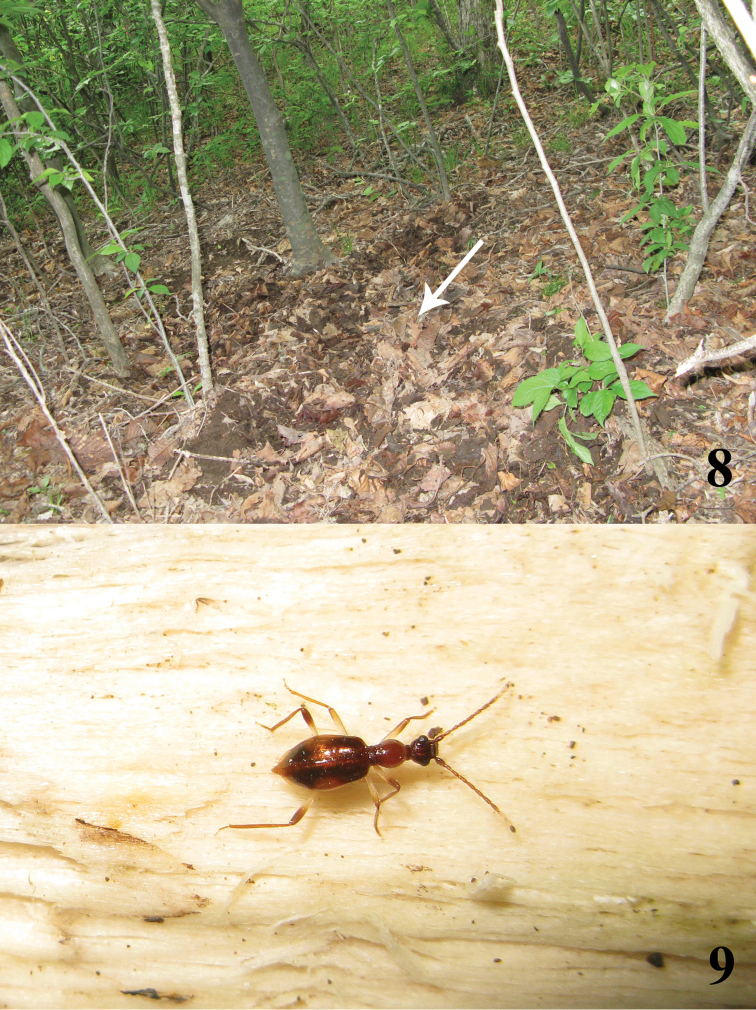
**8** Habitat in Longwangshan **9** *Brathinus satoi* moving on wet log.

Most specimens were collected by sifting leaf litter along a stream in the forest, sometimes even along the bed of temporary brooks (Fig. 8). Two individuals were observed actively moving on the underside of a wet log lying close to a tiny stream (Fig. 9). In the past ten years, many collecting trips were made to Longwanshan from the middle of April to the beginning of October, covering all altitudes of the area in each trip (300–1500m), and the collections show that the activity period of the adults is during late April through May at the higher altitudes of the area, above 1000m.

## Supplementary Material

XML Treatment for Brathinus satoi
